# Supplementary material for: Engineered reversible inhibition of SpyCatcher reactivity enables rapid generation of bispecific antibodies
Source: Nat Commun. 2024 Jul 15;15:5939. doi: 10.1038/s41467-024-50296-y (PMC11251281; doi:10.1038/s41467-024-50296-y)
Supplement: Supplementary file 1 — Supplementary Information [file 41467_2024_50296_MOESM1_ESM.pdf]

Supplementary Information

Engineered Reversible Inhibition of SpyCatcher Reactivity Enables Rapid Generation of Bispecific Antibodies

Christian Hentrich[1, *], Mateusz Putyrski[1, *], Hanh Hanuschka[1], Waldemar Preis[1], Sarah-Jane Kellmann[1], Melissa Wich[1], Manuel Cavada[1], Sarah Hanselka[1], Victor S. Lelyveld[2,3], Francisco Ylera[1, +]

[1]Bio-Rad AbD Serotec GmbH, Anna-Sigmund-Str. 5, 82061 Neuried, Germany

[2]Department of Molecular Biology, Massachusetts General Hospital

[3]Department of Genetics, Harvard Medical School, Boston, MA, USA.

[*]These authors contributed equally

[+]Correspondence should be addressed to francisco_ylera@bio-rad.com

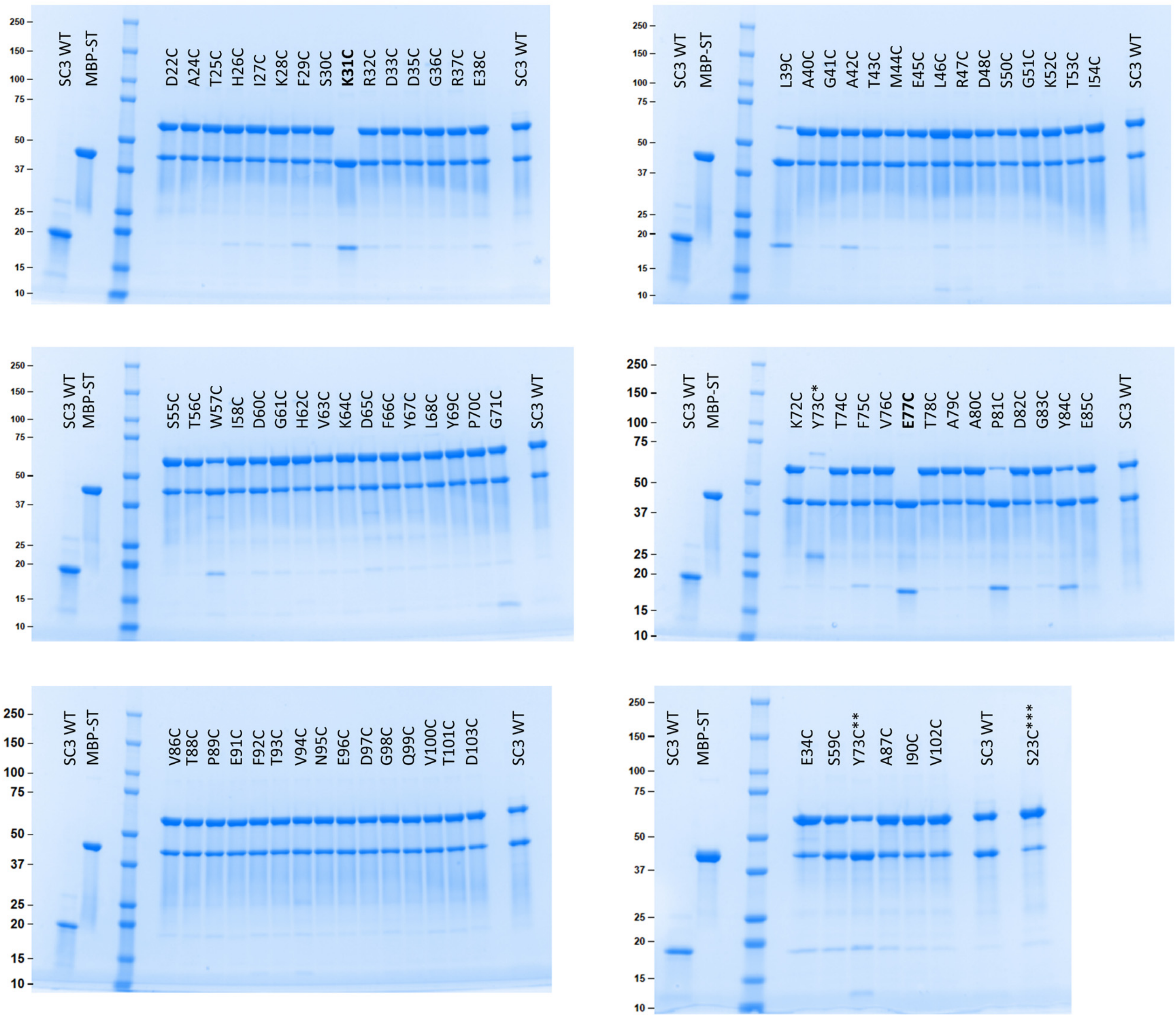

Supplementary Figure 1: SpyCatcher003 cysteine scan – reactivity of cysteine mutants

SDS-PAGE analysis of 81 SpyCatcher003 (SC3) mutants and wildtype after coupling with MBP-SpyTag002 (MBP-ST). Input SpyCatcher003 wildtype and MBP-SpyTag002 are in two leftmost lanes of each gel. Reaction conditions: 4 µM SpyCatcher003 variant, 6 µM MBP-SpyTag002, 1 hour reaction time in PBS. The position of each mutant is indicated on the gel. The first purification of SpyCatcher003 Y72C (labeled with *) did not contain sufficient SpyCatcher and was therefore repeated (**). The S23C mutant (***) contains two additional cysteines, at the N-terminus and an S49C mutation. Reactive and catalytic residues are emphasized in bold print. The screening was performed as a single replicate. Numbers on the left are the molecular weight in kilodaltons.

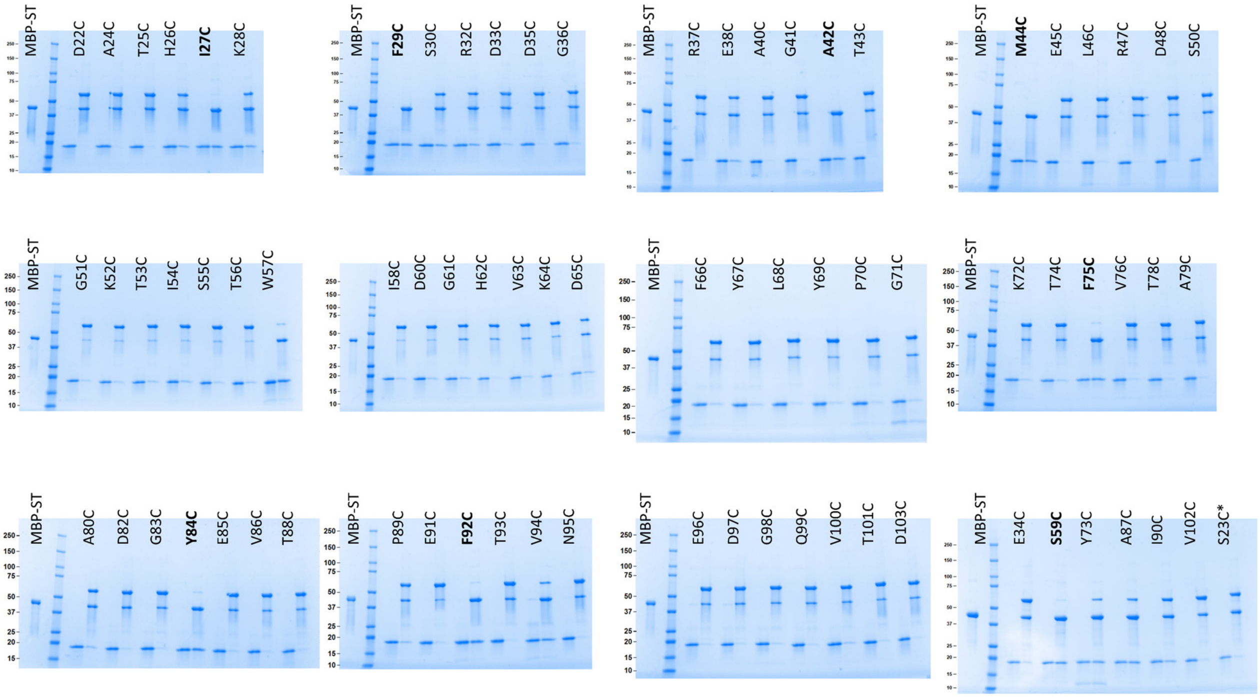

Supplementary Figure 2: SpyCatcher003 cysteine scan – reactivity after modification with TNB

SDS-PAGE analysis of 79 different SpyCatcher003 cysteine mutants modified with Ellman's reagent. Input MBP-SpyTag002 is applied in the leftmost lane of each gel. Each SpyCatcher mutant is loaded in two sequential lanes, as input and as product of reaction with MBP-SpyTag002 (4 µM SpyCatcher003 mutant, 6 µM MBP-SpyTag002, 1.5 hours reaction time). Each pair of lanes is labeled with the mutant name. SpyCatcher003 mutants for which TNB modification led to inhibition of SpyTag coupling are highlighted in bold. The S23C mutant (*) contains two additional cysteines, at the N-terminus and an S49C mutation. The screening was performed as a single replicate. Numbers on the left are the molecular weight in kilodaltons.

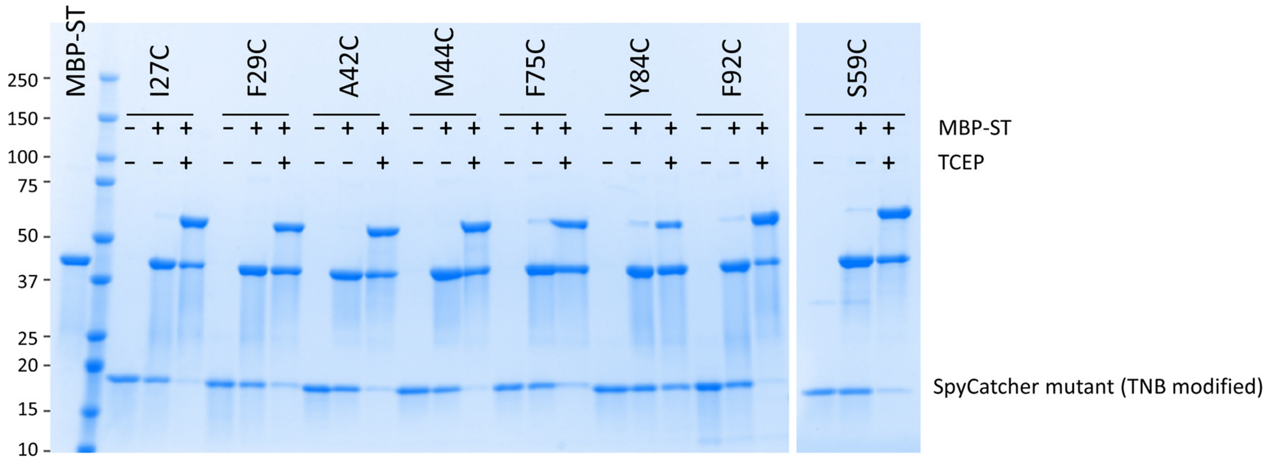

Supplementary Figure 3: SpyCatcher003 cysteine scan – reversibility of inhibition by reduction

SDS-PAGE analysis of 8 SpyCatcher003 cysteine mutants for which SpyTag reactivity was inhibited by modification with TNB. Each mutant is loaded in three sequential lanes, as input in its TNB modified form, mixed with MBP-SpyTag002, and mixed with MBP-SpyTag002 in presence of 10 mM TCEP. The name of the respective SpyCatcher003 mutant is indicated above the corresponding gel lanes. Reaction conditions of SpyTag coupling: 4 µM SpyCatcher003 mutant, 6 µM MBP-SpyTag002, 1.5 hours reaction time in PBS. Experiment performed as a single replicate. Numbers on the left are the molecular weight in kilodaltons.

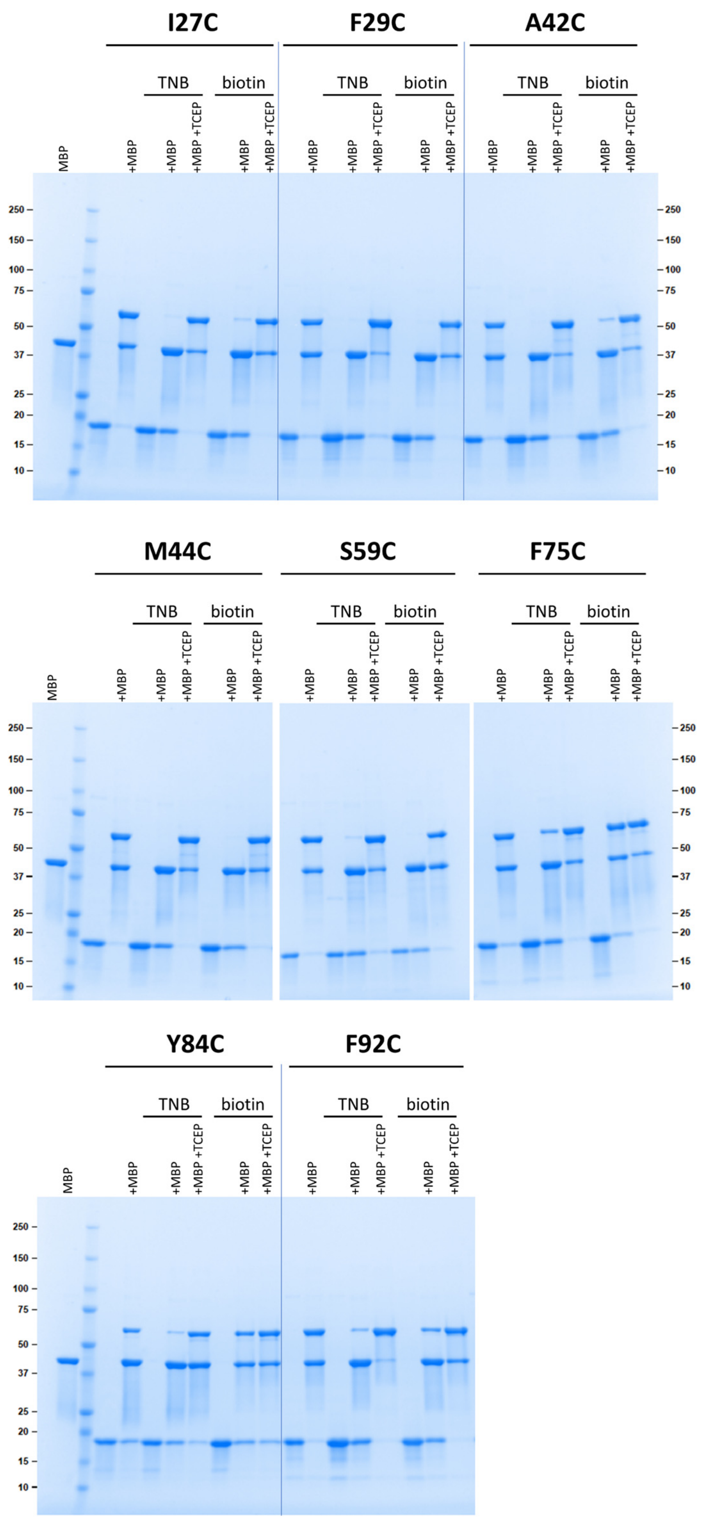

SDS-PAGE analysis of 8 SpyCatcher003 cysteine mutants without disulfide modification, modified with TNB and modified with HPDP-biotin, conjugated with MBP-SpyTag002 in the absence or presence of reducing agent. Each mutant is loaded in 8 sequential lanes, precise reaction conditions are indicated above each lane. Dependent on the reaction conditions, following concentrations of reactants were used: 4 µM SpyCatcher003 mutant, 6 µM MBP-SpyTag002 (abbreviated as MBP in the gel labels), 5 mM TCEP. In all cases reaction time was 1.5 hours. Experiment performed as a single replicate. Numbers on the left are the molecular weight in kilodaltons.

Supplementary Figure 4: Effectiveness of reversible inhibition with TNB and HPDP-biotin

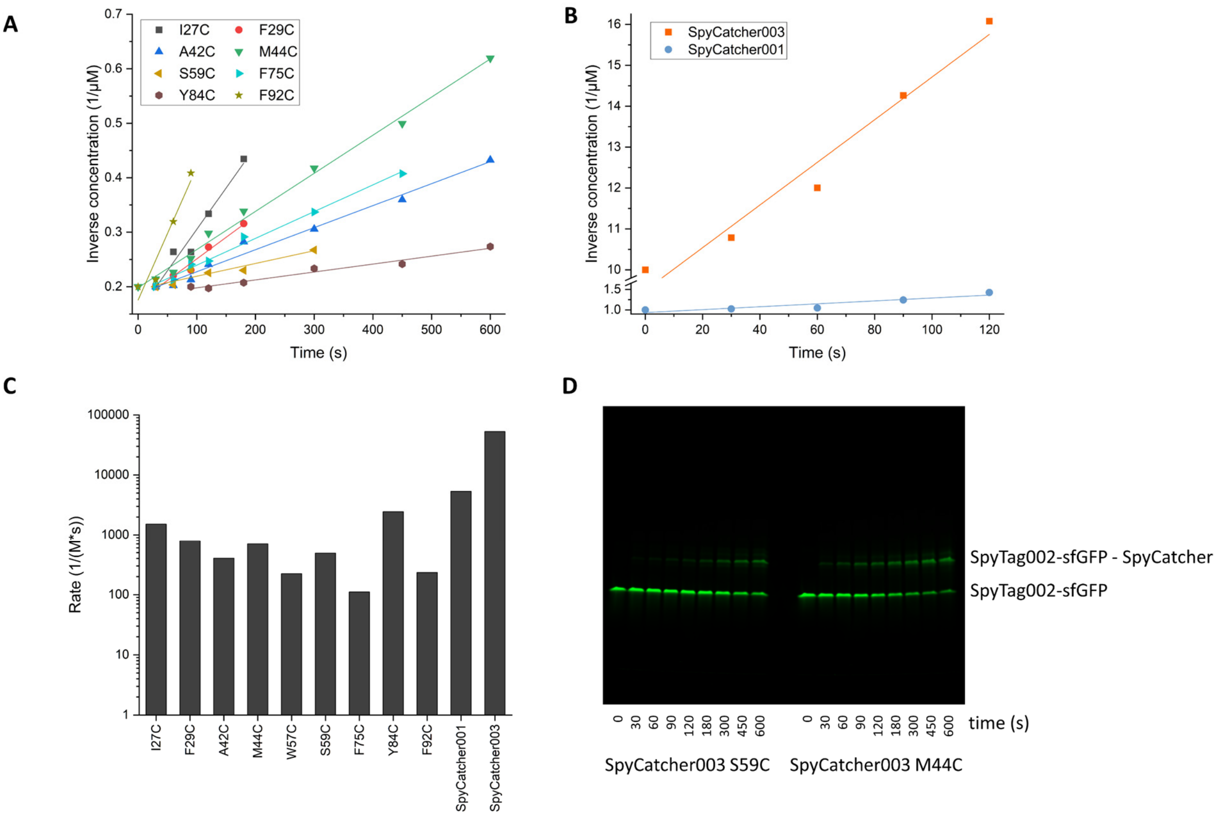

Supplementary Figure 5: Unmodified SpyLock kinetics

Kinetics of SpyLocks and SpyCatcher001/003 were determined by monitoring the reaction with SpyTag002-sfGFP on SDS PAGE and direct imaging of in-gel fluorescence. Reactions were stopped by incubation with SDS sample buffer at 50°C as described[1]. A,B: Inverse SpyTag002-sfGFP concentration plotted over time for SpyLocks (A) and SpyCatchers (B) to measure second order rate constants. C: Measured rate constant derived from linear fits. Numeric values for reaction rates and fit errors are also listed in Suppl. Table 1. D: Example of a typical gel used to monitor the reaction. Image recorded in the fluorescein channel on a ChemiDoc MP (Bio-Rad). Measured rate constants are also provided in Suppl. Table 1. Experiment performed as a single replicate. Source data are provided as a Source Data file.

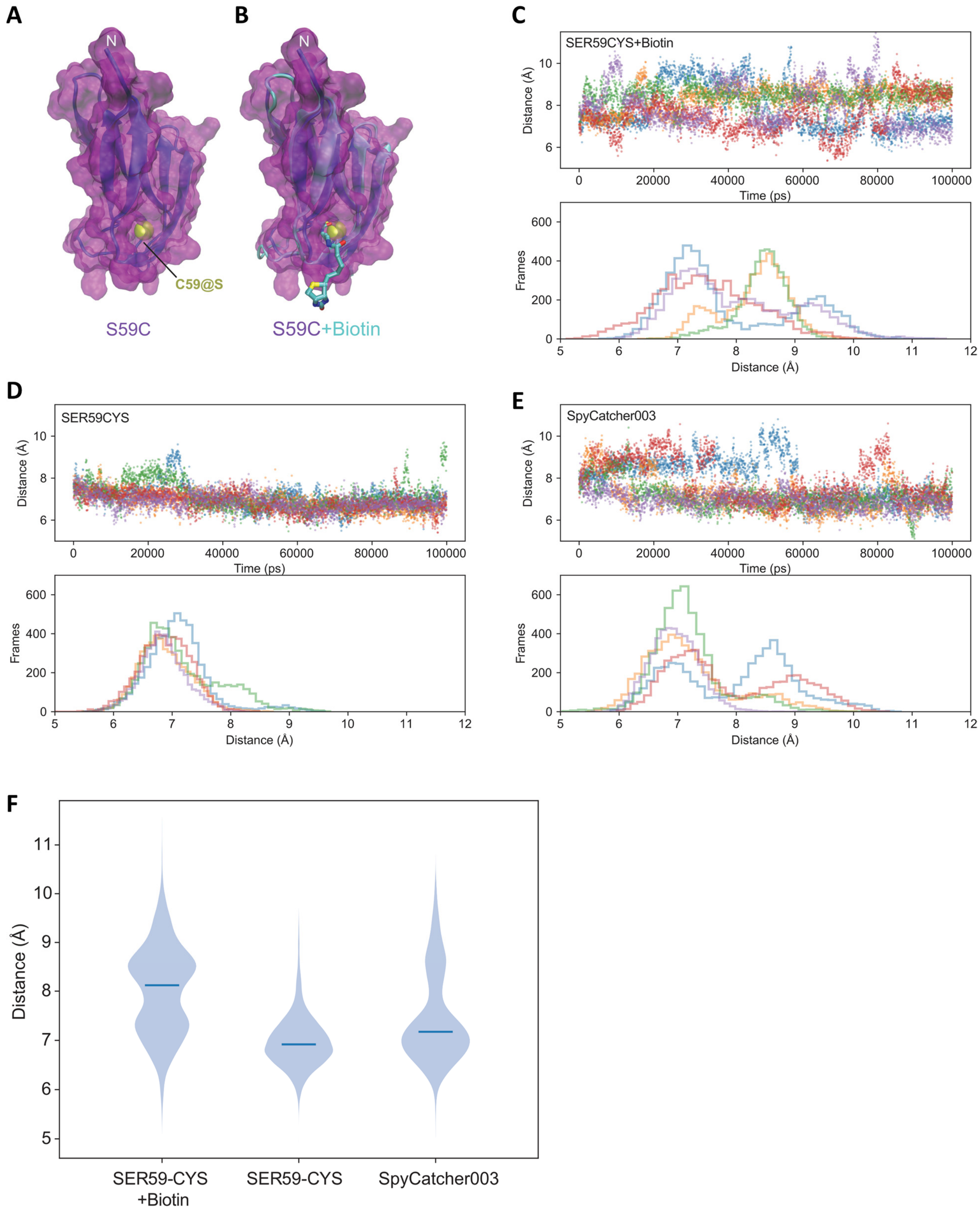

Supplementary Figure 6: MD of the S59C mutant

A: The solvent-excluded surface of the S59C mutant (pink) and the van der Waals radius of the cysteine side chain S atom (yellow) superimposed on the backbone ribbon in the unbound state (purple). B: The surface in panel A overlaid on the biotinylated model at ~10 ns (300 K), showing the conformation of the disulfide-linked modification extending through the pore that is solvent-accessible in the unmodified Cys59 surface. C-E: MD simulations of the destabilizing effect of biotin

modification on S59C mutant SpyCatcher003 at elevated temperature. Replicate simulation trajectories (n = 5 each, colored differently) over 100 ns at 350 K of the indicated system in the presence of bound SpyTag (top: trajectories of C-alpha distance between Lys31 and Asp7 of SpyTag; bottom: histogram of C-alpha distances in each trajectory). F: Violin plot of the data in panels C-E, with median C-alpha distance indicated.

Source data are provided as a Source Data file.

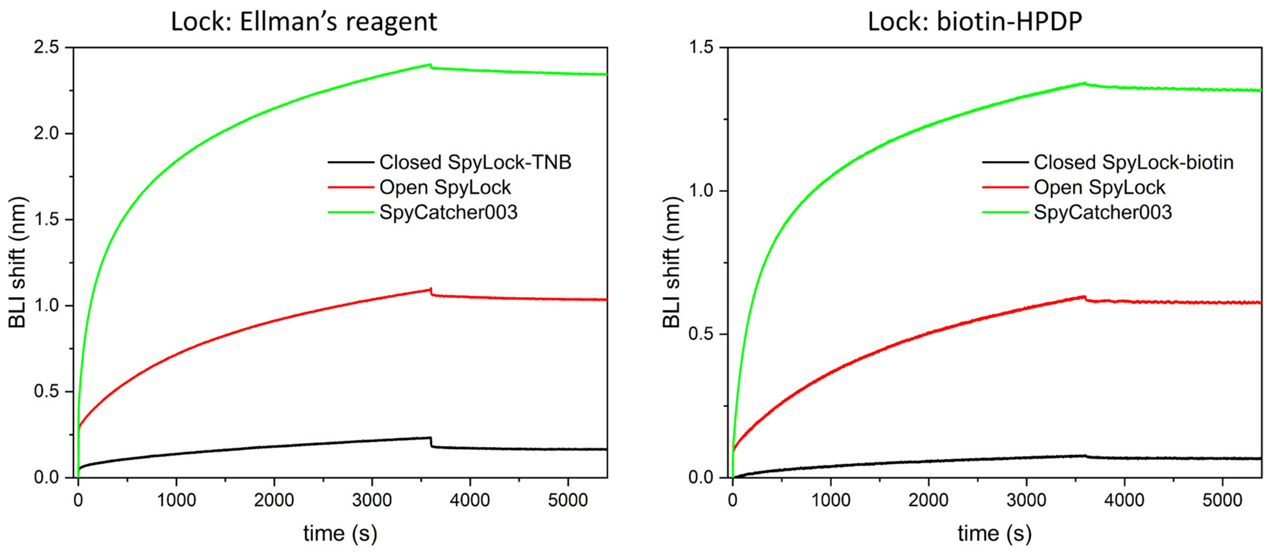

Supplementary Figure 7: Reaction of open and closed SpyLock with SpyTag in BLI

BLI sensorgrams of SpyLock S59C or SpyCatcher003 binding to immobilized biotin-SpyTag002. Association until 3600 seconds, followed by dissociation. Left: DTNB-modified SpyLock. Right: Biotin-modified SpyLock. Representative traces from two independent experiments. Source data are provided as a Source Data file.

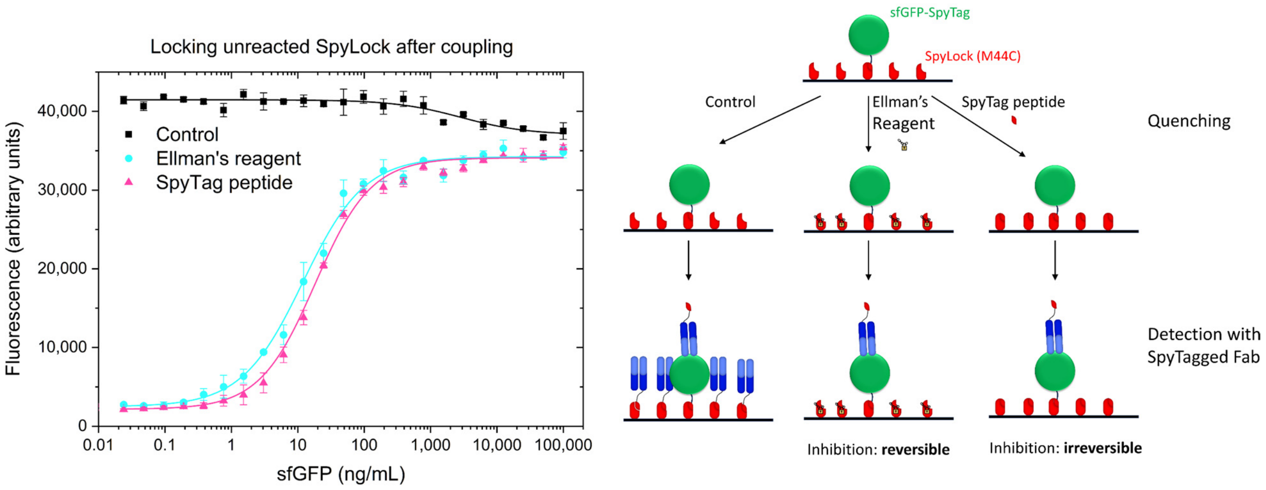

Supplementary Figure 8: Inhibiting SpyLock during an ELISA assay

Left: Titration ELISA showing the detection of sfGFP immobilized via SpyLock M44C via a SpyTagged anti-GFP Fab and anti-Fab-HRP. Without inactivating the SpyLock reactivity prior to detection, the assay cannot be performed successfully. Inactivation via Ellman's reagent and SpyTag003 peptide has the same efficiency. Error bars are standard deviations of triplicate measurements. Right: Scheme of the three assay conditions. Source data are provided as a Source Data file.

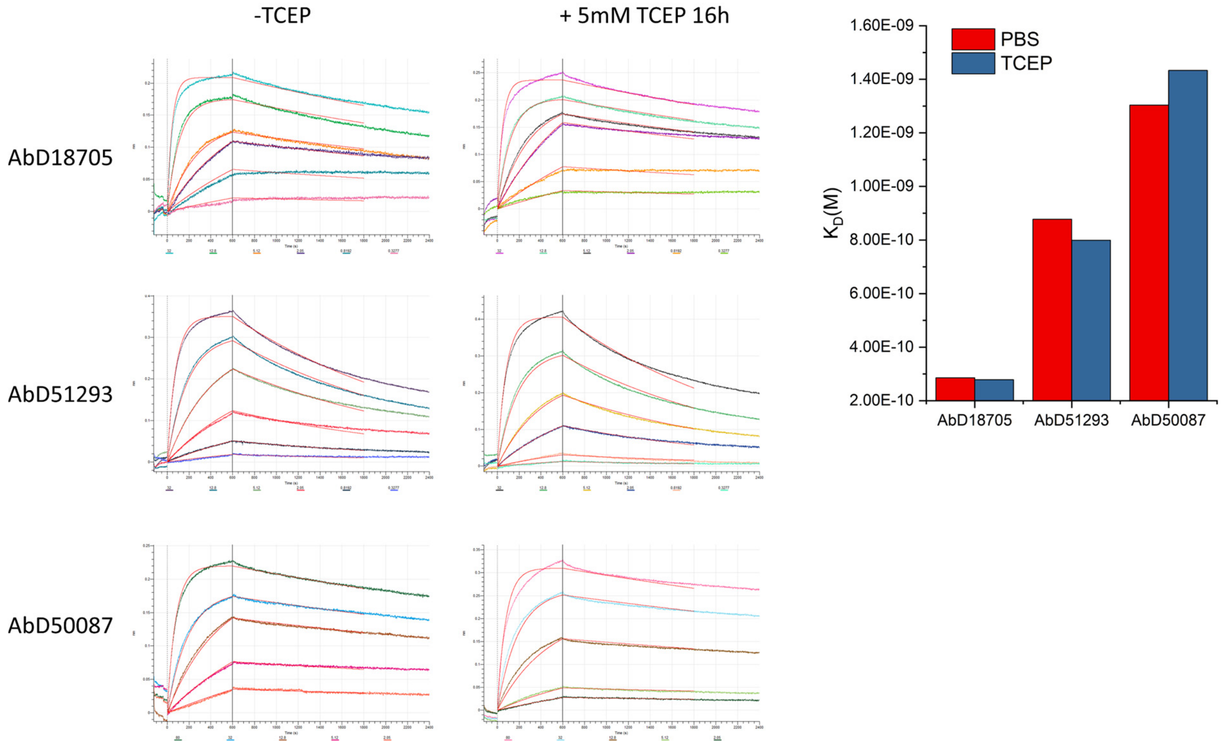

Supplementary Figure 9: Influence of overnight incubation with 5 mM TCEP on Fabs

Left: BLI sensorgrams of three different antibodies against GFP incubated for 16 hours without or with TCEP. Y-axis is BLI shift in nm. Numbers underlined in color are the legend for the BLI traces, indicating the antibody concentration in nM. Fits are colored red.

Right: measured affinities to mGFP-biotin. Measured values together with fit errors are also provided in Suppl. Table 2.

Source data are provided as a Source Data file.

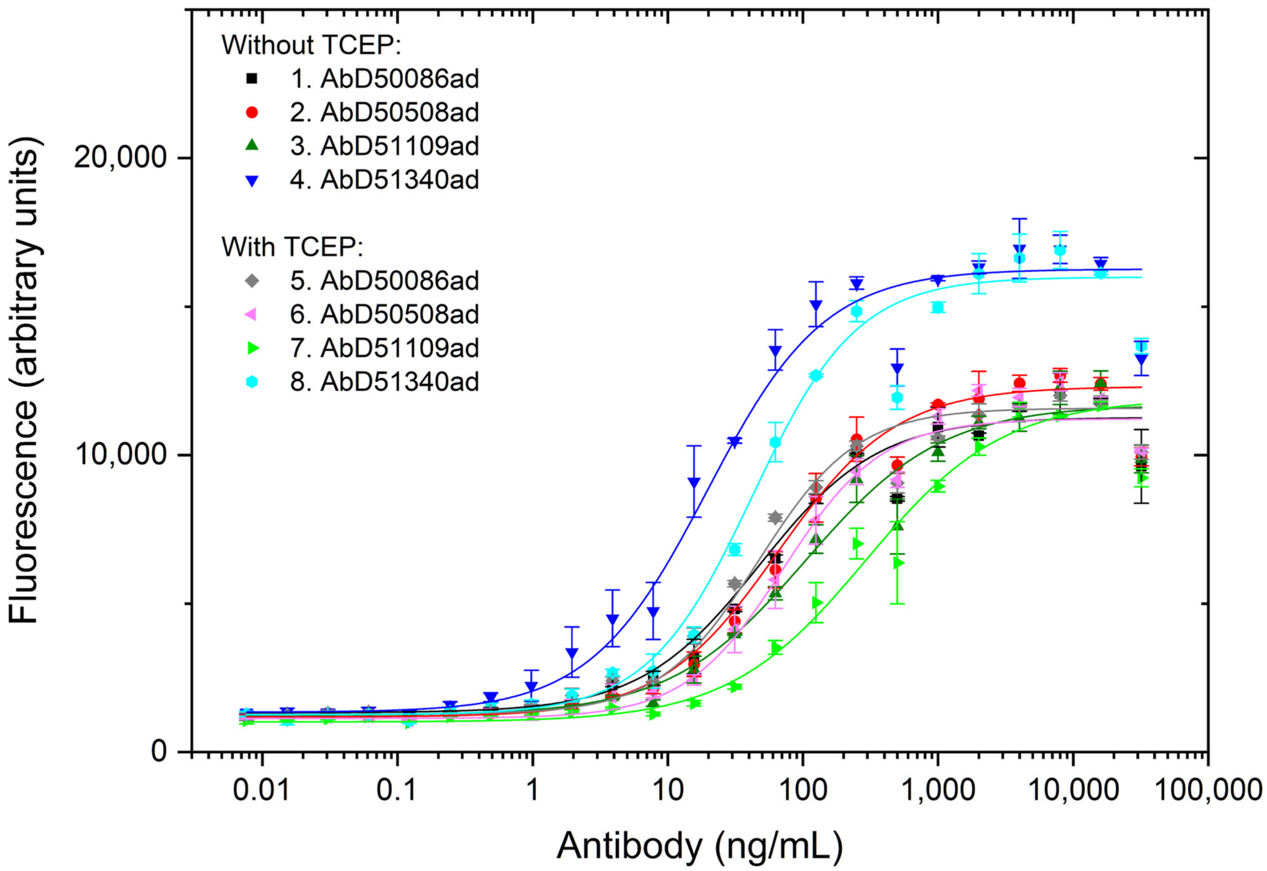

Supplementary Figure 10: Effect of long-term TCEP incubation on antigen binding

Titration ELISA of 4 different anti-GFP Fabs: AbD50086ad, AbD50508ad, AbD51109ad, AbD51340ad, after 1 week of incubation at 4°C with or without 5mM TCEP. mGFP was coated, anti-GFP Fabs were titrated and detection was performed with anti-Fab-HRP. Error bars represent standard deviation of 2 measurements. Logistic fit was applied. Source data are provided as a Source Data file.

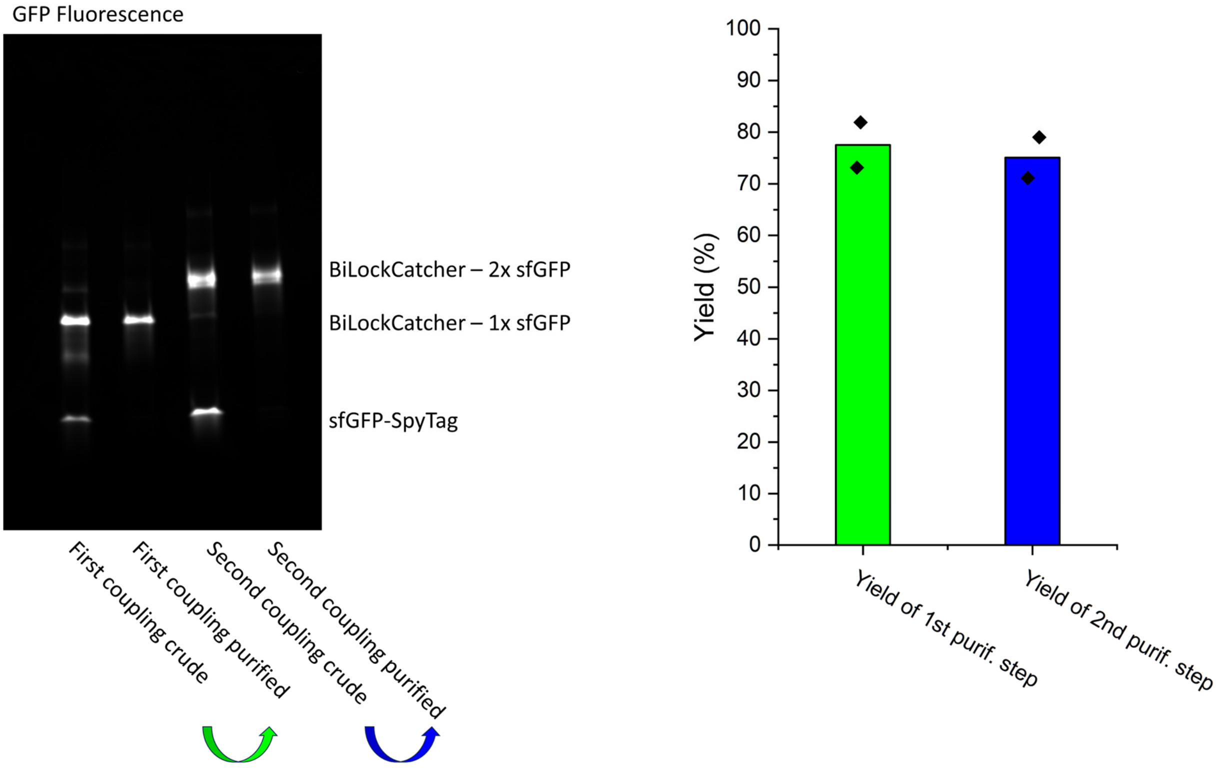

Supplementary Figure 11: Yield of BiLockCatcher purification

Left: SDS PAGE showing sfGFP fluorescence of the first and second purification step of the BiLockCatcher reaction (purity protocol), both couplings performed with SpyTag002-sfGFP. Experiment was performed twice independently, representative gel shown. Right: Yield of the product band of both purification steps quantified from in-gel fluorescence. Bars are the mean of two independent experiments. Source data are provided as a Source Data file.

A

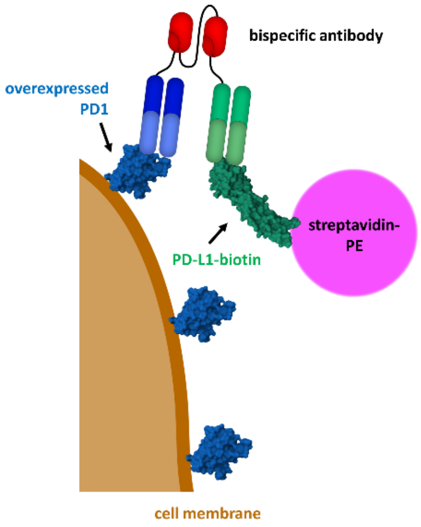

B

Symbol	Antigen	International nonproprietary name
A	PD-L1	atezolizumab
B	PD-L1	avelumab
C	PD-L1	durvalumab
1	PD-1	cemiplimab
2	PD-1	dostarlimab
3	PD-1	nivolumab
4	PD-1	pembrolizumab

C

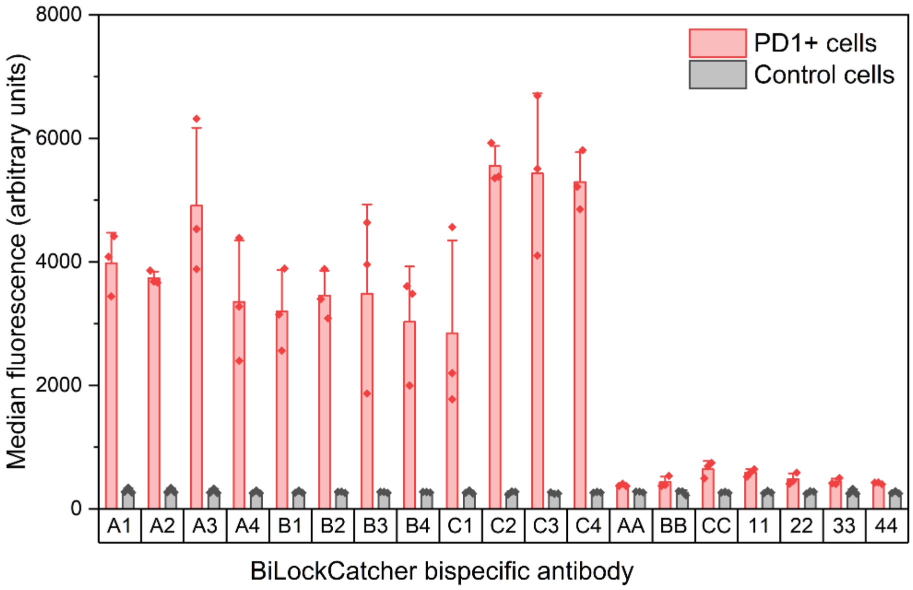

Supplementary Figure 12: Bispecific cell staining assay

A) Schematic representation of the bispecific cell staining assay: PD-1-overexpressing HKB11 cells are incubated consecutively with BiLockCatcher-based bispecific antibodies or control antibodies, PD-L1-biotin and then stained with streptavidin-PE and analyzed by flow cytometry.

B) List of therapeutic anti-PD-L1 (antibodies A-C) and anti-PD-1 (antibodies 1-4) antibodies used in the cell staining assay. All antibodies were expressed as SpyTagged Fabs and used for construction of BiLockCatcher bispecific reagents. To exemplify the nomenclature of the obtained products: antibody ‘B4’ denotes bispecific avelumab-BiLockCatcher-pembrolizumab whereas ‘33’ denotes monospecific nivolumab-BiLockCatcher-nivolumab.

C) Median fluorescence from cell staining of PD-1-overexpressing cells or control cells. Error bars are standard deviations of triplicate measurements.

Source data are provided as a Source Data file.

BiLockCatcher-biotin:

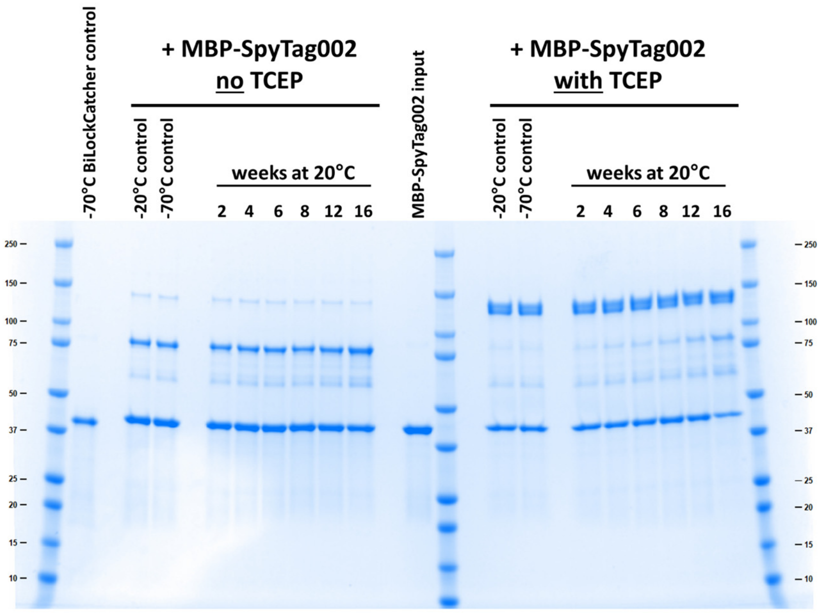

BiLockCatcher-TNB:

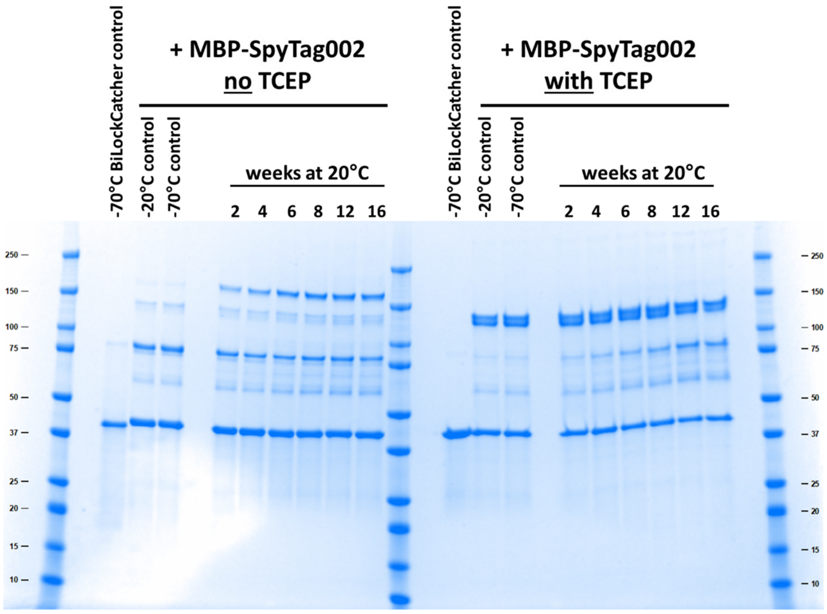

Supplementary Figure 13: Stability of BiLockCatcher

BiLockCatcher modified with HPDP-biotin or DTNB was incubated for 16 weeks at 20°C and samples were taken at regular intervals and stored at -70°C. Then, BiLockCatcher samples collected over 16 weeks were used for coupling with MBP-SpyTag002, which was performed either in absence or in presence of 20 mM TCEP. The coupled samples were applied on a non-reducing SDS-PAGE gel. BiLockCatcher samples stored for 16 weeks at -20°C and -70°C were used as controls. Reaction conditions: 5 µM BiLockCatcher, 12 µM MBP-SpyTag002, 1 hour at RT. Unconjugated BiLockCatcher

and MBP-SpyTag002 input samples were also included. Upper panel: samples of BiLockCatcher blocked with HPDP-biotin, lower panel: BiLockCatcher samples blocked with DTNB. Experiment performed as a single replicate. Numbers on the left and right are the molecular weight in kilodaltons.

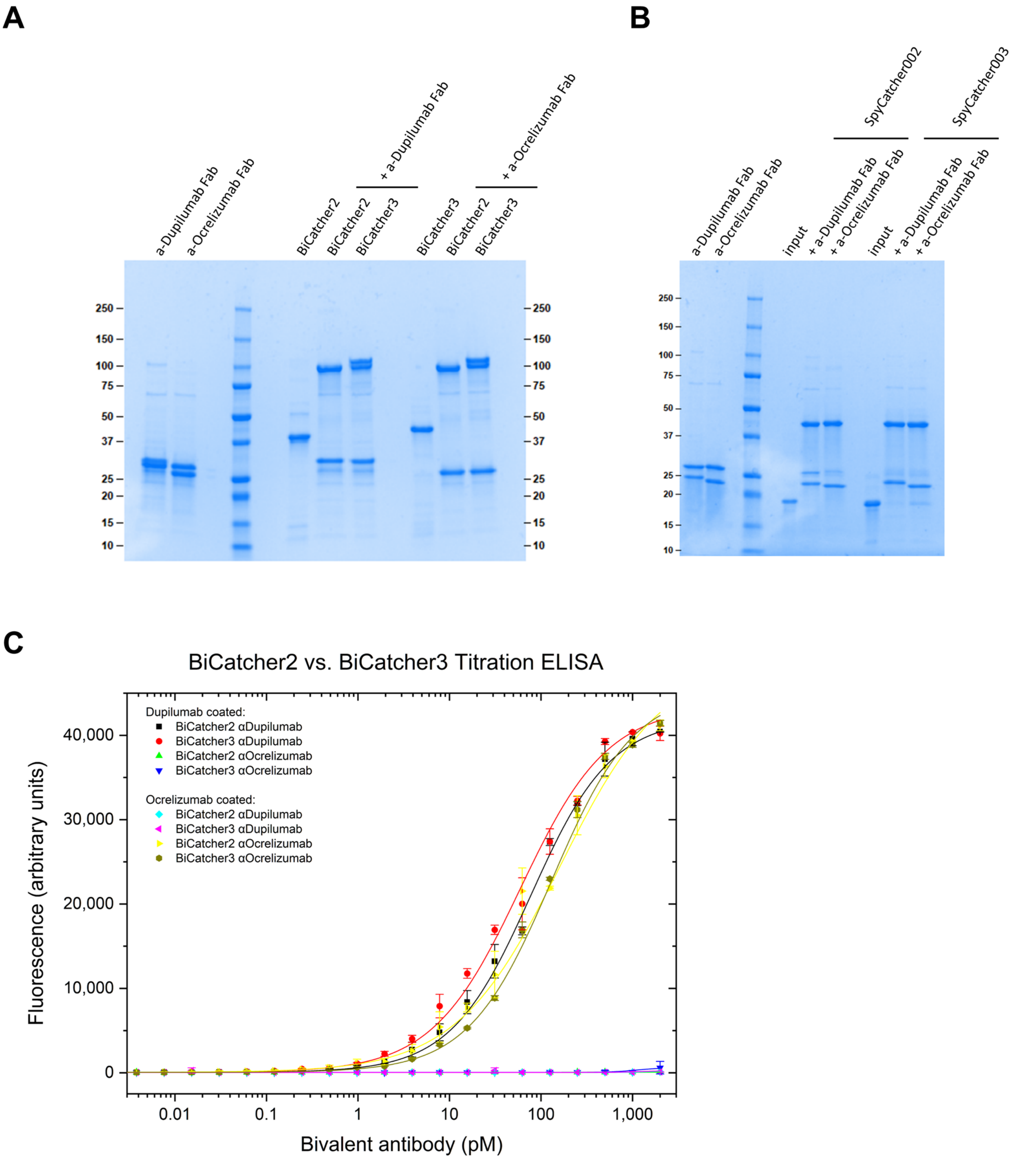

Supplementary Figure 14: Double band formation of BiCatcher3 after SpyTag coupling

We observed an interesting aspect of producing bivalent constructs when using BiLockCatcher based on SpyCatcher003. The BiLockCatcher construct as well as BiCatcher3 coupling products migrated as double bands in SDS-PAGE when fully coupled, while fully coupled monomeric SpyCatcher003 does not form double bands. Bivalent constructs based on SpyCatcher001 and 002 do not exhibit this phenomenon. While this observation is at first glance reminiscent of the deamidation occurring in SpyCatcher001 and 2[2], a simple posttranslational modification cannot be the cause here, as the monovalent starting materials and monovalent coupling products do not exhibit double bands in SDS-PAGE. Given the limited number of amino acid differences between SpyCatcher002 and

SpyCatcher003 — only five in total — one of these substitutions is likely responsible for the observed occurrence of double bands in dimeric constructs, which otherwise show a single band when reacting as individual monomeric proteins. In any case, there is no observable difference in affinity between bivalent constructs based on SpyCatcher002 and 003, therefore the difference is irrelevant for antibody screening.

A) Non-reducing SDS-PAGE gel of coupling reaction products of BiCatcher2 (SpyCatcher002-SpyCatcher002 fusion) and BiCatcher3 (SpyCatcher003-SpyCatcher003 fusion) with two different Fabs along input controls. Experiment performed as a single replicate. Numbers on the left are the molecular weight in kilodaltons.

B) Reducing SDS-PAGE gel of coupling reaction products of monomeric SpyCatcher002 and monomeric SpyCatcher003 with two different Fabs along input controls. Experiment performed as a single replicate. Numbers on the left are the molecular weight in kilodaltons.

C) Titration ELISA comparing the sensitivity and specificity of BiCatcher2 and BiCatcher3 bivalent constructs generated with anti-dupilumab and anti-ocrelizumab Fabs. Dupilumab or ocrelizumab were coated (2 µg/mL), as indicated. Stated bivalent constructs were titrated onto the plate and HRP-conjugated anti-His-Tag antibody (Bio-Rad, MCA5995P, 1:2000) was used for detection. Error bars represent standard deviation of 2 measurements. Logistic fit was applied. Source data are provided as a Source Data file.

Supplementary Table 1: Reaction rates with SpyTag002-sfGFP (Suppl. Fig. 5)

Errors are fit errors from linear regression.

**SpyCatcher**	**Rate constant ($M^{-1}s^{-1}$)**
I27C	1.5E+3 ± 1.7E+2
F29C	7.9E+2 ± 7.3E+1
A42C	4.0E+2 ± 2.2E+1
M44C	7.0E+2 ± 2.1E+1
S59C	4.9E+2 ± 2.1E+1
F75C	1.1E+2 ± 1.2E+1
Y84C	2.4E+3 ± 4.8E+2
F92C	2.3E+2 ± 4.1E+1
SpyCatcher001	5.3E+3 ± 5.3E+2
SpyCatcher003	5.3E+4 ± 3.1E+3

Supplementary Table 2: anti-GFP Fab affinities without or with TCEP incubation (Suppl. Fig. 9)

Fab	K_D in PBS (M)	Fit Error	K_D in PBS+5mM TCEP (16h) (M)	Fit Error
AbD18707	2.9E-10	7.9E-13	2.8E-10	6.9E-13
AbD51293	8.8E-10	1.8E-12	8.0E-10	3.6E-12
AbD50087	1.3E-09	1.7E-12	1.4E-09	2.3E-12

Supplementary References

1. Keeble AH*, et al.* Approaching infinite affinity through engineering of peptide-protein interaction. *Proc Natl Acad Sci U S A*, (2019).

2. Hentrich C*, et al.* Periplasmic expression of SpyTagged antibody fragments enables rapid modular antibody assembly. *Cell Chem Biol* **28**, 813-824 e816 (2021).
