# Supplementary material for: Engineered reversible inhibition of SpyCatcher reactivity enables rapid generation of bispecific antibodies
Source: Nat Commun. 2024 Jul 15;15:5939. doi: 10.1038/s41467-024-50296-y (PMC11251281; doi:10.1038/s41467-024-50296-y)
Supplement: Supplementary file 3 — Reporting summary [file 41467_2024_50296_MOESM3_ESM.pdf]

nature portfolio

Corresponding author(s): Francisco Ylera

Last updated by author(s): Jun 20, 2024

Reporting Summary

Nature Portfolio wishes to improve the reproducibility of the work that we publish. This form provides structure for consistency and transparency in reporting. For further information on Nature Portfolio policies, see our Editorial Policies and the Editorial Policy Checklist.

Statistics

For all statistical analyses, confirm that the following items are present in the figure legend, table legend, main text, or Methods section.

n/a	Confirmed	
☐	☒	The exact sample size (*n*) for each experimental group/condition, given as a discrete number and unit of measurement
☒	☐	A statement on whether measurements were taken from distinct samples or whether the same sample was measured repeatedly
☒	☐	The statistical test(s) used AND whether they are one- or two-sided<br>*Only common tests should be described solely by name; describe more complex techniques in the Methods section.*
☒	☐	A description of all covariates tested
☒	☐	A description of any assumptions or corrections, such as tests of normality and adjustment for multiple comparisons
☐	☒	A full description of the statistical parameters including central tendency (e.g. means) or other basic estimates (e.g. regression coefficient) AND variation (e.g. standard deviation) or associated estimates of uncertainty (e.g. confidence intervals)
☒	☐	For null hypothesis testing, the test statistic (e.g. *F*, *t*, *r*) with confidence intervals, effect sizes, degrees of freedom and *P* value noted<br>*Give P values as exact values whenever suitable.*
☒	☐	For Bayesian analysis, information on the choice of priors and Markov chain Monte Carlo settings
☒	☐	For hierarchical and complex designs, identification of the appropriate level for tests and full reporting of outcomes
☒	☐	Estimates of effect sizes (e.g. Cohen's *d*, Pearson's *r*), indicating how they were calculated

Our web collection on statistics for biologists contains articles on many of the points above.

Software and code

Policy information about availability of computer code

Data collection	NanoDSF: Nanotemper PR.Thermocontrol v2.1.2<br>Plate reader: Tecan i-control 3.7.3.0<br>Luminescence: Tecan Sparkcontrol 1.2<br>Gels: Bio-Rad Image Lab 6.1.0
Data analysis	Calculations: MS Excel 365<br>Gels: Bio-Rad Image Lab 6.1.0<br>Flow: De Novo FCS Express 7.16.0035<br>Diagrams: OriginLab Origin 2022 9.9.0.220<br>Modeling: Alphafold v2.3.2 (simplified), OpenMM 8.1.0<br>Structure illustrations: VMD 1.9.4a55, UCSF Chimera 1.15

For manuscripts utilizing custom algorithms or software that are central to the research but not yet described in published literature, software must be made available to editors and reviewers. We strongly encourage code deposition in a community repository (e.g. GitHub). See the Nature Portfolio guidelines for submitting code & software for further information.

Data

Policy information about availability of data

All manuscripts must include a data availability statement. This statement should provide the following information, where applicable:

- Accession codes, unique identifiers, or web links for publicly available datasets
- A description of any restrictions on data availability
- For clinical datasets or third party data, please ensure that the statement adheres to our policy

The authors declare that the data supporting the findings of this study are available within the paper and the supplementary information. Source data are provided with this paper. Should any raw data files be needed in another format they are available from the corresponding author upon reasonable request. The previously published structure 4MLI can be found here: 4MLI [http://doi.org/10.2210/pdb4MLI/pdb]. Uniprot entry Q15116 can be found here: Q15116 [https://www.uniprot.org/uniprotkb/Q15116/entry]

Research involving human participants, their data, or biological material

Policy information about studies with human participants or human data. See also policy information about sex, gender (identity/presentation), and sexual orientation and race, ethnicity and racism.

Reporting on sex and gender	n/a
Reporting on race, ethnicity, or other socially relevant groupings	n/a
Population characteristics	n/a
Recruitment	n/a
Ethics oversight	n/a

Note that full information on the approval of the study protocol must also be provided in the manuscript.

Field-specific reporting

Please select the one below that is the best fit for your research. If you are not sure, read the appropriate sections before making your selection.

☒ Life sciences ☐ Behavioural & social sciences ☐ Ecological, evolutionary & environmental sciences

For a reference copy of the document with all sections, see nature.com/documents/nr-reporting-summary-flat.pdf

Life sciences study design

All studies must disclose on these points even when the disclosure is negative.

Sample size	We did not perform statistical tests
Data exclusions	No data was excluded.
Replication	With the exception of large screening experiments and rate measurements, all experiments were performed in replicates, indicated by error bars, or a representative gel is shown from at least two independent experiments.
Randomization	n/a
Blinding	n/a

Reporting for specific materials, systems and methods

We require information from authors about some types of materials, experimental systems and methods used in many studies. Here, indicate whether each material, system or method listed is relevant to your study. If you are not sure if a list item applies to your research, read the appropriate section before selecting a response.

Materials & experimental systems

n/a	Involved in the study
☐	☒ Antibodies
☐	☒ Eukaryotic cell lines
☒	☐ Palaeontology and archaeology
☒	☐ Animals and other organisms
☒	☐ Clinical data
☒	☐ Dual use research of concern
☒	☐ Plants

Methods

n/a	Involved in the study
☒	☐ ChIP-seq
☐	☒ Flow cytometry
☒	☐ MRI-based neuroimaging

Antibodies

Antibodies used	Primary antibodies: Bio-Rad. anti-Fab-HRP: Bio-Rad (Order # STAR126P, polyclonal, 1:1000). a-Penta-His: Bio-Rad (Order # MCA5995P, clone ABD2.2.20, 1:2000). Ocrelizumab, Dupilumab: Pharmacy.
Validation	All Bio-Rad primary antibodies were verified by full Sanger sequencing of the expression plasmid and QC ELISA of produced antibodies on target and control antigens.

Eukaryotic cell lines

Policy information about cell lines and Sex and Gender in Research

Cell line source(s)	Jurkat-Lucia TCR-hPD-1 effector cells and Raji-APC-hPD-L1 antigen presenting cells: Invivogen (#rajkt-hpd1)<br>HKB11: Bio-Rad
Authentication	Cell lines were not authenticated.
Mycoplasma contamination	Eurofins Mycoplasma tests were regularly performed.
Commonly misidentified lines<br>(See ICLAC register)	n/a

Plants

Seed stocks	n/a
Novel plant genotypes	n/a
Authentication	n/a

Flow Cytometry

Plots

Confirm that:

☐ The axis labels state the marker and fluorochrome used (e.g. CD4-FITC).

☒ The axis scales are clearly visible. Include numbers along axes only for bottom left plot of group (a 'group' is an analysis of identical markers).

☐ All plots are contour plots with outliers or pseudocolor plots.

☐ A numerical value for number of cells or percentage (with statistics) is provided.

Methodology

Sample preparation	Suspension cells were harvested from cell culture flasks (50 mL)
Instrument	Bio-Rad ZE5

Software	De Novo FCS Express 7.16.0035
Cell population abundance	n/a
Gating strategy	Gating was performed on living cells (based on DAPI stain) and on single cells (based on FSC-A vs FSC-H)

☐ Tick this box to confirm that a figure exemplifying the gating strategy is provided in the Supplementary Information.
